# Prevention of mood disorder after stroke: a randomised controlled trial of problem solving therapy versus volunteer support

**DOI:** 10.1186/s12883-019-1349-8

**Published:** 2019-06-14

**Authors:** Kate Hill, Allan House, Peter Knapp, Carrie Wardhaugh, John Bamford, Andy Vail

**Affiliations:** 1 Leeds Institute of Health Sciences, University of Leeds, Worsley Building (Rm 11.57), Clarendon Way, Leeds, LS2 9NL UK; 2 Department of Health Sciences and the Hull York Medical School, University of York, York, YO10 5DD UK; 3 Centre for Clinical Brain Sciences, National CJD Research and Surveillance Unit, Bryan Matthews Building, Western General Hospital, Crewe Road, Edinburgh, EH4 2XU UK; 4 Leeds Teaching Hospitals Trust, Great George Street, Leeds, LS1 3EX UK; 5 The University of Manchester, Oxford Rd, Manchester, M13 9PL UK

**Keywords:** Stroke, Cerebrovascular accident, Depression, Mood disorders, Problem solving, Cognitive therapy, Prevention

## Abstract

**Background:**

Mood disorder after stroke is common but drug and psychosocial treatments have been assessed with disappointing results. Preventing mood disorder from developing in the first place could be a better approach and might reduce the need for pharmacotherapy in this predominantly older patient group. We used a brief problem-solving therapy and evaluated its effect in reducing mood disorder in the 12 months after stroke.

**Methods:**

A 3-group, parallel, randomised controlled trial. Four hundred fifty patients with stroke were randomised within 1 month of hospital admission to problem-solving therapy from a psychiatric nurse, non-specific support given by volunteers or treatment-as-usual. Follow up took place at 6 and 12 months after stroke.

Standardised measures of mood (Present State Examination, GHQ-28), cognitive state (mini-mental state examination) and function (Barthel ADL index, Frenchay Activities Index) were taken at baseline, 6 and 12 months after randomisation. Satisfaction with care was recorded at follow up.

**Results:**

At 6 months, all psychological and activity measures favoured problem-solving therapy. At 12 months, patients in the problem-solving therapy group had significantly lower GHQ-28 scores and lower median Present State Examination symptom scores. There were no statistically significant differences in activity. The problem-solving therapy group were more satisfied with some aspects of care.

**Conclusions:**

The results are encouraging and suggest it is possible to prevent mood disorder in stroke patients using a psychological intervention. The differences between the groups at 12 months may indicate a sustained impact of psychological therapies, by comparison with non-specific support.

**Trial registration:**

ISRCTN: ISRCTN33773710 Registered: 23/01/2004 (Retrospectively).

## Background

Stroke patients are a predominantly older group and around 50% of acute stroke survivors have residual major physical or cognitive deficits. Given the need for patients to cope with the complex physical and social sequelae of stroke, the demands of recovery and rehabilitation, and the risk of recurrent stroke, it is not surprising that mood disorder, usually manifesting as anxiety or depression after stroke, is common. The consequences of unreconciled emotional distress can be reduced quality of life and impaired progress in physical and social rehabilitation. A number of drug and psychosocial treatments have been assessed in clinical trials but the results have been disappointing. Drug trials have suggested a role for antidepressants in both treatment and prevention, but the trials are generally of poor quality and do not provide sufficient information to judge their true costs and benefits [1, 2] Psychosocial interventions have similar methodological problems [3]. The latter are popular with patients but there is conflicting evidence for their effectiveness in either treating or preventing anxiety and depression [4, 5].

Nonetheless, psychosocial interventions may have a role in preventing mood disorder after stroke, as recent reviews have concluded, and there is still a need for more trials in this area [6, 7].

## Aims and objectives

This study aimed to evaluate the effect of a brief psychological treatment: problem-solving therapy for reducing mood disorder in the 12 months after stroke [8]. We used two comparison groups: treatment-as-usual, and an attention control group that received non-specific support given by volunteers. Outcomes were measured quantitatively by a widely-used self-report questionnaire, the 28-item General Health Questionnaire (GHQ-28) [9] and by scores derived from a standardised psychiatric interview the Present State Examination: Short Form (PSE) [10]. Our secondary hypotheses were that patients in the problem-solving therapy group would be less likely to have a diagnosable depressive disorder at follow up, would have better social function and would be more satisfied with their care.

## Methods

### Study design and participants

A 3-group, parallel, randomised controlled trial.

### Sample

Patients admitted with first ever or recurrent stroke (diagnosed on clinical history and signs, supplemented by CT brain scan when ordered by clinician) to hospitals in Leeds and Bradford. We were notified of stroke admissions in the two Leeds trusts by the Leeds Stroke Database. In Bradford we contacted admitting wards each week.

#### Inclusion criteria

Adults admitted to hospital with first ever or recurrent stroke (other than subarachnoid haemorrhage) within the past month; who were local residents and able to give written consent.

#### Exclusion criteria

Patients who were: too ill to interview within 1 month of stroke; unable to participate through impaired speech, cognition or use of English; living in a residential home; had a serious concurrent illness, which was likely to dominate the pattern of care or were participating in another rehabilitation trial.

### Randomisation and masking

The trial had three arms: problem-solving therapy from a psychiatric nurse, non-specific support given by volunteers or treatment-as-usual. We screened all notifications of stroke admissions, and after obtaining verbal consent for the initial assessment we undertook a baseline interview and a Mini-Mental State Examination (MMSE) [11]. Randomisation was third party, after a telephone call from a research interviewer to a remote trials office. Random allocation was generated by computer in the trials office in blocks of 15, stratified by admitting NHS trusts. Eligible patients were then asked to give written consent to the treatment to which they had been randomised, and to be followed up at 6 and 12 months. If the patient refused the intervention we asked them to consent to follow-up. Patients were sent a letter confirming their participation and giving the name of their volunteer or nurse, as appropriate, and the planned follow-up dates. In this way, patients were not aware that their treatment was being randomly allocated, and did not know that other patients were receiving a different treatment. This design, which is a variant of Zelen’s procedure [12], was approved by the three local research ethics committees that reviewed the study.

Patients were not masked to their allocation as this is clearly impossible to achieve with this type of intervention. However, as a result of the randomisation procedure patients were unaware of other treatment allocations.

### Interventions and comparators

#### Problem-solving therapy

This short-term therapy was delivered in the patient’s home, after discharge, by one of two Community Psychiatric Nurses employed specifically for the study. The aim was to improve the patient’s problem solving skills, so that the patient feels he or she is taking control of coping. Improved coping skills should result in reduced psychological distress and rates of depression. The therapist followed a manual in helping the patient to identify and prioritise problems, set goals and identify solutions to the problems, choose and try a plausible solution and then re-assess in the light of the results. The therapy had six sessions: identifying stroke related problems including gaps in knowledge about stroke; identifying non-stroke problems; identifying available external resources; identifying personal coping resources; looking at the problem-solving process, and summarising the process. The therapy has been described in more detail elsewhere [13]. The six sessions were planned to be given in 6 h, about a fortnight apart, with the patient doing ‘homework’ between each meeting. One benefit of a manual-based therapy is that the therapist can give more or less time to each session by monitoring the patient’s progress. The therapy was adaptable to be used with or without a carer present. The therapists received training and regular clinical supervision from a specialist liaison psychiatrist (AH). The therapy was not given if the patient was discharged to residential care or remained in hospital 6 months after stroke.

#### Non-specific support

One of 47 volunteers recruited to the study was assigned to provide talking (non-specific) support. The volunteers attended a training meeting that focussed on the consequences of stroke. More than half the volunteers had personal or family experience of stroke. We asked the volunteer to visit the patient 6–8 times and paid travel expenses. For practical reasons the volunteers began their visits to the patient soon after randomisation, sometimes before the patient was discharged.

#### Treatment as usual

No additional effort was made, during the course of the trial, to enhance routine psychiatric care in stroke services. At the time of the trial, no stroke service in Leeds or Bradford had dedicated clinical psychology or psychiatry time available for the treatment of mood disorders associated with stroke, although referral was possible from all services for mental health assessment and treatment of cases identified by stroke staff as requiring specialist care.

### Outcome assessment

A trained interviewer obtained personal and social details, and undertook the following assessments during the initial interview:

The *Mini-Mental State Examination (MMSE)* [11]*,* a brief screen for cognitive dysfunction. It is scored 0–30 with higher scores indicating better function.

The *Barthel index* [14], which assesses activities of daily living skills. It is scored 0–20, with higher scores indicating greater abilities. The patient’s self-reported pre-stroke and post-stroke abilities were assessed.

The *Frenchay Activities Index* [15] is a measure of social function, scored 0–45, with higher scores indicating greater function. It is broader in scope than the Barthel index, including items to assess frequency of shopping, travel, hobbies, etc. The patient’s self-reported pre-stroke activities were assessed.

The *Present State Examination: short form (PSE)* [10] is a semi-structured, standardised psychiatric interview from which is derived an index of definition. The index has a range 1–7, with a score of > 5 indicating probable psychiatric disorder; we gave each of these cases a psychiatric diagnosis according to the research criteria of ICD-10.

The *General Health Questionnaire* (*GHQ-28*) [9] is a measure of psychological distress scored 0–28, with higher scores indicating greater distress. Scores on the GHQ can be used to identify probable psychiatric disorder: for neurological in-patients the threshold is > 12 and among outpatients the threshold is > 9 and above.

The patients also completed an adapted version of a scale to assess satisfaction with aspects of care given in hospital and after discharge [16].

Follow up assessments took place in the patient’s home with an interviewer who was not informed of the treatment allocation of the patient. Patients who received problem-solving were asked by the therapist not to reveal their allocation. To test the extent of unmasking of outcome assessors, we asked the interviewer to guess the allocation of 127 patients seen at 12 months.

Primary assessments were PSE and GHQ-28 at 12 months. Secondary assessments were: the Barthel index, the Frenchay Activities Index, patient satisfaction, medication use, and contacts with health and social services at 12 months, and all the above at 6 months.

### Sample size calculation

The sample size of 450 was calculated to give 80% power to detect a 2-point difference in GHQ score, including an estimate of 20% lost to follow-up, through death and refusals, randomly distributed between the groups. We based the sample size calculation for the study on producing a 2-point change because no minimally clinically important difference has been defined for the GHQ since it is designed primarily as a screening tool.

### Statistical analysis

Prior to starting the study we planned to undertake two forms of analysis: in the first (Phase 1) we planned to compare the outcome data for all three groups at 6 & 12 months to test variation between them. In the second (Phase 2) we planned to compare problem-solving therapy with the other 2 groups to test its benefit.

Our methods of analysis included the Kruskal-Wallis (non-parametric) analysis of variance to examine ordinal scales in Phase 1. Other measures were dichotomised and analysed using logistic regression in Phase 2 of our plan. We used therapy as the ‘baseline’ group, since we planned the trial to compare it with both treatment-as-usual and non-specific support, similar to that provided in previous studies [17].

## Results

### Recruitment

**Fig. 1:**
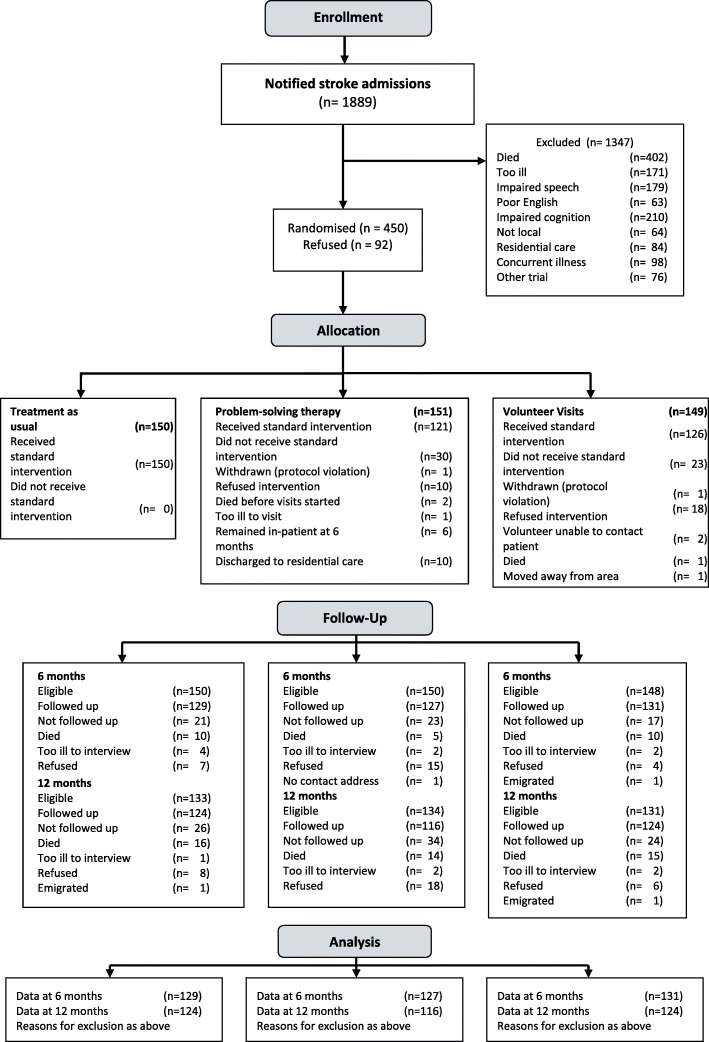
CONSORT Flow Diagram

We received notification of almost 1900 stroke admissions over 23 months (see Fig. 1), of which 542 (28.5%) patients were eligible for inclusion. Of this total 450 (83.0%) patients agreed to the baseline interview and were randomised. Two patients were randomised in error: one who had not suffered a stroke and another who also suffered dementia. These patients were withdrawn. The random allocation resulted in similar numbers and distribution of baseline variables in the three groups (see Table 1). The three methods of assessing psychiatric disorder produced similar rates: approximately one fifth of patients were identified as having a mood disorder within 1 month of stroke.

**Table 1:** Baseline data for all patients and the three randomised groups

Baseline variable N (%) or Median (IQR)	All patients	Problem solving therapy	Volunteer support	Treatment as usual
Female	207 (46%)	66 (44%)	76 (51%)	65 (43%)
Age	72 (65–79)	71 (65–79)	72 (64–78)	74 (68–80)
Previous stroke	94 (21%)	36 (24%)	26 (18%)	32 (21%)
Home owner	249 (56%)	78 (52%)	86 (58%)	85 (57%)
Lived alone pre-stroke	175 (39%)	64 (43%)	52 (35%)	59 (39%)
No named carer	46 (10%)	17 (11%)	14 (9%)	15 (10%)
In any paid work (pre-stroke)	131 (29%)	36 (24%)	45 (30%)	50 (33%)
Initial hospital stay (days)	27 (12–60)	26 (13–64)	27 (12–71)	26 (15–55)
Pre-stroke Barthel < 20	132 (29%)	51 (34%)	43 (29%)	38 (25%)
Pre-stroke Frenchay	27 (21–34)	26 (11–33)	29 (22–34)	29 (20–34)
Post-stroke Barthel	15 (9–18)	14 (9–18)	14.5 (9–19)	15 (9–18)
MMSE score	26 (23–28)	26 (23–28)	26 (23–28)	26 (23–27)
GHQ-28 total score	5 (2–9)	6 (2–10)	5 (2–10)	5 (2–9)
GHQ-28 score ≥ 12	85 (19%)	29 (19%)	27 (18%)	29 (20%)
PSE total symptom score	5 (2–9)	5 (3–9)	5 (2–8)	4 (2–7)
PSE Index of definition ≥5	102 (23%)	34 (23%)	35 (24%)	33 (22%)
Major depressive episode	100 (22%)	36 (24%)	34 (23%)	30 (20%)
Any depressive episode	145 (32%)	51 (34%)	51 (34%)	43 (29%)

At the 12 month interview we were able to interview 124/133 (93.2%) eligible patients in treatment as usual, 116/134 (86.6%) in problem-solving therapy, and 124/131 (94.6%) in volunteer support (see Fig. 1).

### Contact with intervention

Thirty patients randomised to problem-solving therapy (19.9%) did not receive any therapy intervention. For the majority (19) this was because they were unable to do so (see Fig. 1), but 10 patients refused the intervention either immediately after randomisation or after discharge when the therapist phoned to make the first appointment and one was withdrawn. Remaining patients received between 1 and 10 sessions (median 5) during the 4 months after stroke. Twenty-three patients (15.4%) randomised to support did not receive any volunteer visits. The commonest reason was patient refusal, often immediately after randomisation. Patients received between 1 and 42 volunteer contacts in the first 12 months after stroke (median 6) and many volunteers maintained patient contact beyond 6–8 visits and after we had stopped paying their travel expenses.

### Functional and psychological scores at 6 and 12 months

Patients randomised to problem-solving therapy had non-significantly lower Barthel and Frenchay scores compared to the volunteer support and treatment as usual groups at 6 months. At 12 months, patients in the problem-solving group had lower GHQ scores, with a median score 2 points lower than the treatment as usual group and one point lower than the volunteer support group. When GHQ scores were converted into caseness scores this difference was no longer significant. There was also evidence of differences between the three groups in their Barthel or Frenchay scores similar to those observed at 6 months. Patients in the problem-solving group had lower scores but these differences were non-significant. See Table 2.

**Table 2:** Functional and psychological scores at 6 and 12 months

Variable Scores at 6 months median (IQR)	Problem solving therapy	Volunteer support	Treatment as usual
Barthel Index	17 (12–20)	18 (13–20)	18 (15–20)
Frenchay Activities Index	10 (3–20)	12 (3–23)	12 (5–23)
GHQ-28 score	4 (2–8)	4 (2–9)	5 (2–9)
PSE total symptom score	4 (2–8)	6 (2–9)	5.5 (2–10)
Variable Scores at 12 months median (IQR)	Problem solving therapy	Volunteer support	Treatment as usual
Barthel Index	17 (12–20)	18 (13.5–20)	18 (14–20)
Frenchay Activites Index	9.5 (4–21)	12 (5–24)	14.5 (3.5–26)
GHQ-28 score	3 (1–6)	4 (1–8)	5 (2–8)
PSE total symptom score	4 (2–7)	5 (2–9)	5 (2–9)

### Outcomes

The psychological scores were converted to caseness (a PSE index of definition > 5; a GHQ score > 9 or a major depression diagnosis) we found that the problem-solving therapy group had lower levels of major depression and fewer patients had an index of definition of 5 or above at both 6 and 12 months. Odds ratios and 95% confidence intervals were calculated but the differences did not reach statistical significance.

There was some evidence of differences between the three groups in satisfaction with hospital care, with patients randomised to treatment as usual being the least satisfied. Patients randomised to problem-solving were more satisfied than patients who received treatment as usual with three aspects of care after discharge. Odds ratios (95% CI) were calculated but only three items were significantly different at 6 months: the hospital staff attended to my needs; satisfaction with support to help cope with feelings about stroke and satisfaction with interest shown in worries and concerns of patient since stroke. At 12 months only satisfaction with information received about voluntary organisations was significantly better in the problem-solving therapy group. See Tables 3 and 4.

**Table 3:** Psychology, activity and proportion expressing dissatisfaction at 6 months after stroke

Variable	Problem solving therapy n (%)	Volunteer support n (%) OR (95% CI)	Treatment as usual n (%) OR (95% CI)
GHQ score ≥ 9,	28 / 126 (22)	33 / 127 (26)	37 / 126 (29)
		1.2 (0.69, 2.2)	1.4 (0.82, 3.1)
PSE index of definition ≥5	23 / 126 (18)	31 / 128 (24)	34 / 124 (27)
		1.4 (0.78, 2.6)	1.7 (0.93, 3.1)
Major depression	23 / 126 (18)	34 / 128 (27)	35 / 124 (28)
		1.6 (0.89, 2.9)	1.8 (0.97, 3.2)
Any depression	37 / 126 (29)	42 / 128 (33)	47 / 124 (38)
		1.2 (0.72, 2.1)	1.5 (0.87, 2.5)
I have been treated with kindness and respect by the hospital staff, number dissatisfied	2 / 121 (2)	7 / 125 (6)	1 / 125 (0.8)
		3.5 (0.72, 17)	0.48 (0.04, 5.4)
The hospital staff attended well to my personal needs, number dissatisfied	4 / 121 (3.3)	12 / 125 (10)	3 / 125 (2)
		3.1 (0.97, 9.9)	0.72 (0.16, 3.3)
I felt able to talk to the hospital staff about any problems, number dissatisfied	13 / 120 (11)	12 / 124 (10)	11 / 125 (9)
		0.88 (0.38, 2.0)	0.79 (0.34, 1.8)
I received all the information I wanted about my stroke, number dissatisfied	25 / 121 (21)	30 / 125 (24)	21 / 125 (17)
		1.2 (0.66, 2.2)	0.77 (0.41, 1.5)
The doctors have done everything they can to make me well again, number dissatisfied	7 / 121 (6)	10 / 124 (8)	6 / 124 (5)
		1.4 (0.52, 3.9)	0.83 (0.27, 2.5)
I am happy with the recovery I have made since my illness, number dissatisfied	24 / 121 (20)	25 / 125 (20)	22 / 123 (18)
		1.0 (0.54, 1.9)	0.88 (0.46, 1.7)
I am satisfied with the treatment the therapists (e.g. occupational, physio, speech) have given me, number dissatisfied	13 / 121 (11)	14 / 125 (11)	17 / 124 (14)
		1.0 (0.47, 2.3)	1.3 (0.61, 2.8)
I was given enough information about allowances & services, number dissatisfied	17 / 114 (15)	18 / 123 (15)	25 / 120 (21)
		0.98 (0.48, 2.0)	1.5 (0.76, 3.0)
Things were well prepared for my return home, number dissatisfied	13 / 114 (11)	18 / 123 (15)	15 / 121 (12)
		1.3 (0.62, 2.9)	1.1 (0.50, 2.4)
I am satisfied with the hospital outpatient services, number dissatisfied	12 / 116 (10)	12 / 124 (10)	13 / 121 (11)
		0.93 (0.40, 2.2)	1.0 (0.45, 2.4)
The ambulance service is good and reliable, number dissatisfied	10 / 115 (9)	6 / 122 (5)	9 / 119 (8)
		0.54 (0.19, 1.5)	0.86 (0.34, 2.2)
I get enough support from meals on wheels, home help, etc, number dissatisfied	12 / 112 (11)	10 / 124 (8)	14 / 121 (12)
		0.73 (0.30, 1.8)	1.1 (0.48, 2.5)
I am satisfied with the service from my GP, number dissatisfied	10 / 116 (9)	13 / 125 (10)	9 / 121 (7)
		1.2 (0.52, 2.9)	0.85 (0.33, 2.2)
I am satisfied with the support to help me cope with my feelings about the stroke, number dissatisfied	9 / 115 (8)	18 / 124 (14)	27 / 120 (22)
		2.0 (0.86, 4.6)	3.4 (1.5, 7.6)
I am satisfied with the interest shown in my worries and concerns since the stroke, number dissatisfied	11 / 115 (10)	22 / 124 (17)	27 / 120 (22)
		2.0 (0.94, 4.4)	2.7 (1.3, 5.8)
I was given enough information about voluntary organisations, number dissatisfied	19 / 116 (16)	24 / 124 (19)	35 / 120 (29)
		1.2 (0.63, 2.4)	2.1 (1.1, 3.9)
I am satisfied that my family were encouraged to be involved in my care, number dissatisfied	9 / 112 (8)	6 / 120 (5)	8 / 119 (7)
		0.60 (0.21, 1.7)	0.82 (0.31, 2.2)

**Table 4:** Psychology, activity and proportion expressing dissatisfaction at 12 months after stroke

Variable	Problem solving therapy n (%)	Volunteer support n (%) OR (95% CI)	Treatment as usual n (%) OR (95% CI)
GHQ ≥9	17 / 116 (15)	26 / 124 (21)	28 / 123 (23)
		1.5 (0.79, 3.0)	1.7 (0.88, 3.3)
PSE index of definition ≥5	22 / 115 (19)	31 / 124 (25)	30 / 124 (24)
		1.4 (0.76, 2.6)	1.3 (0.72, 2.5)
Major depression	21 / 115 (18)	33 / 124 (27)	31 / 124 (25)
		1.6 (0.88, 3.0)	1.5 (0.80, 2.8)
Any depression	32 / 115 (28)	38 / 124 (31)	38 / 124 (31)
		1.1 (0.64, 1.9)	1.1 (0.63, 1.9)
I have been treated with kindness and respect by the hospital staff, number dissatisfied	1 / 111 (0.9)	3 / 115 (3)	2 / 121 (2)
		2.9 (0.30, 29)	1.8 (0.16, 21)
The hospital staff attended well to my personal needs, number dissatisfied	4 / 111 (4)	6 / 115 (5)	7 / 121 (6)
		1.5 (0.40, 5.4)	1.6 (0.47, 5.8)
I felt able to talk to the hospital staff about any problems, number dissatisfied	9 / 111 (8)	12 / 115 (10)	11 / 121 (9)
		1.3 (0.53, 3.3)	1.1 (0.45, 2.8)
I received all the information I wanted about my stroke, number dissatisfied	13 / 111 (12)	24 / 115 (21)	20 / 121 (16)
		2.0 (0.95, 4.1)	1.5 (0.70, 3.2)
The doctors have done everything they can to make me well again, number dissatisfied	4 / 111 (4)	8 / 114 (7)	7 / 121 (6)
		2.0 (0.59, 6.9)	1.6 (0.47, 5.8)
I am happy with the recovery I have made since my illness, number dissatisfied	20 / 111 (18)	26 / 115 (23)	28 / 120 (23)
		1.3 (0.69, 2.5)	1.4 (0.73, 2.6)
I am satisfied with the treatment the therapists (e.g. occupational, physio, speech) have given me, number dissatisfied	13 / 111 (12)	9 / 115 (8)	13 / 120 (11)
		0.64 (0.26, 1.6)	0.92 (0.40, 2.1)
I was given enough information about allowances & services, number dissatisfied	12 / 107 (11)	21 / 112 (19)	17 / 119 (14)
		1.8 (0.85, 3.9)	1.3 (0.60, 2.9)
Things were well prepared for my return home, number dissatisfied	9 / 107 (8)	18 / 111 (16)	17 / 118 (14)
		2.1 (0.90, 4.9)	1.8 (0.78, 4.3)
I am satisfied with the hospital outpatient services, number dissatisfied	9 / 107 (8)	19 / 110 (17)	12 / 118 (10)
		2.3 (0.98, 5.3)	1.2 (0.50, 3.0)
The ambulance service is good and reliable, number dissatisfied	7 / 108 (6)	6 / 106 (6)	5 / 118 (4)
		0.87 (0.28, 2.7)	0.6 (0.20, 2.1)
I get enough support from meals on wheels, home help, etc, number dissatisfied	6 / 106 (6)	17 / 107 (16)	12 / 118 (10)
		3.1 (1.6, 8.3)	1.9 (0.68, 5.2)
I am satisfied with the service from my GP, number dissatisfied	7 / 107 (6)	17 / 111 (15)	12 / 117 (10)
		2.6 (1.0, 6.5)	1.6 (0.62, 4.3)
I am satisfied with the support to help me cope with my feelings about the stroke, number dissatisfied	14 / 108 (13)	17 / 111 (15)	19 / 118 (16)
		1.2 (0.57, 2.6)	1.3 (0.61, 2.7)
I am satisfied with the interest shown in my worries and concerns since the stroke, number dissatisfied	13 / 108 (12)	16 / 111 (14)	23 / 117 (20)
		1.2 (0.56, 2.7)	1.8 (0.85, 3.7)
I was given enough information about voluntary organisations, number dissatisfied	13 / 108 (12)	19 / 110 (17)	33 / 116 (28)
		1.5 (0.71, 3.3)	2.9 (1.4, 3.7)
I am satisfied that my family were encouraged to be involved in my care, number dissatisfied	7 / 107 (6)	10 / 108 (9)	11 / 116 (9)
		1.5 (0.53, 4.0)	1.5 (0.56, 4.0)

More patients in the problem-solving group dropped out of the study than the other two groups, mainly due to a higher refusal rate. One possibility is that patients who received less therapy, or who were doing less well in therapy, were more likely to drop out. However, if we assume that the 18 problem-solving patients who refused 12 month follow-up had the same GHQ scores as the mean of followed-up patients in the treatment as usual group, the mean GHQ score in the problem-solving group would have been 4.6 rather than 4.5, with no change in the statistical significance of our result.

The 17 items of the satisfaction scale showed only 1 significant difference across all 3 groups, which is consistent with what would be expected in this number of secondary analyses.

To test the extent of masking we asked the interviewer to guess the allocation of the last 127 patients, after their 12 month interview. The interviewer guessed correctly in 65 (51.2%) patients (kappa = 0.26, *p* < .001), suggesting that some unmasking had occurred. Treatment as usual was the most frequent guess.

## Discussion

There is some evidence from our study that problem-solving therapy can be effective in reducing emotional disorder at 12 months after stroke though the benefits observed are modest. The therapy group had lower GHQ scores at 12 months; and were more satisfied with aspects of their care related to psychological outcomes at 6 months. There was no difference in activity scores; indeed patients in the therapy group were the least active of the 3 groups. It is disappointing that therapy had no effect on social activity, but perhaps not surprising given that our chosen measure for this outcome (the Frenchay Activities Index) is heavily dependent on basic physical abilities. We surmise that patients’ activities were limited more by the disabling effects of their stroke than by their inclination to be socially active.

We chose GHQ as our primary outcome because it is one of the most widely used and validated questionnaires to assess mood and because it has been tested in numerous populations including people with stroke [18]. When we analysed the data on the basis of presence of psychiatric disorder, defined according to research interview, the difference was no longer-significant. One possibility is that the intervention helps milder emotional disturbance but not more severe states. Another interpretation is that our study was under-powered to show a significant reduction in this more stringent test. It is also possible that the continuation of volunteer visits beyond the planned intervention period, something that occurred in a number of cases in our study, diluted any between-groups differences; for example explaining why the lower GHQ scores observed in the therapy group at 6 months did not reach significance.

Unlike many studies of psychological therapy we did not limit recruitment to patients with symptoms of mood disorder. Instead we used a broad recruitment strategy in order to test the effects of the intervention as a preventive treatment. We also thought it unlikely that a brief psychological treatment such as problem solving would have a meaningful effect on patients with an established mood disorder. We decided not to include patients in residential care, who live in environments where it can be difficult to be an active problem solver, and where it would be difficult to deliver the intervention effectively. We also excluded patients with levels of cognitive ability, speech and use of English that would make the delivery of a talking psychological therapy problematic.

We acknowledge that our recruitment strategies limit the generalisability of the results in a wider stroke population but we feel this is justified because the application of problem- solving therapy in practice would most likely be targeted at the community dwelling stroke patient with the potential to regain some degree of independence in daily living. If we assume that in routine practice therapy might be offered to those we excluded because they were in another trial, those who did not live locally and those who refused randomisation, then a total of 682/1487 (46%) stroke survivors might be suitable for such a brief therapy.

Overall the results of this trial are encouraging but nevertheless we recognise that a number of limitations need to be considered. Multiple item testing is a problem that affects the measurement of satisfaction and quality of life measures. This study used a large number of outcome measures thereby increasing the possibility of chance findings. The primary outcome variable was GHQ score and this was significantly different between the three groups, but mood was also assessed by GHQ caseness, total symptom score, PSE index of definition and major depression, at both 6 & 12 months after stroke. However, it is important to note that all the significant differences favoured the therapy group, including a measure of satisfaction of many aspects of hospital and community care.

We also acknowledge the effect of interviewer bias and the extent of unmasking of the outcome interviewers to the patient’s allocation but the influence of this effect on the results should not be overstated given that in almost 50% occasions the interviewer failed to guess the patient’s allocation correctly. Furthermore, the two significant measures: the GHQ and the satisfaction scale, were self-report measures thus lessening the effect of interviewer bias.

The impact of the volunteer visitors is harder to discount as we did not control what they were doing in their intervention. The results of a small diary and focus group study suggest that there was considerable variation between the volunteers, and that the type of intervention they provided was driven by their personal experience of stroke.

## Conclusion

Our trial randomised 150 patients to receive a short-term psychological treatment and demonstrated that problem-solving therapy has potential value for the reduction or prevention of emotional disorders after stroke. Benefits of interventions have two components: the specific effects of the treatment itself and the perception that the therapy is being given. In this study, as a placebo arm was not possible, we controlled for non-specific effects using the volunteer support arm to which a similar number of patients were randomised. Replication is needed to confirm our findings, preferably in multi-centre trials with larger sample sizes, and incorporating some form of process measure in an attempt to gain a better understanding of how patients make use of the therapy intervention.

## Data Availability

The data that support the findings of this study are available from the author upon reasonable request.

## Abbreviations

CI: Confidence interval
GHQ-28: General health questionnaire 28-item
IQR: Interquartile range
MMSE: Mini-Mental State Examination
NHS: National health service
PSE: Present state examination (short form)
